# Remarks on Dialysis

**Published:** 1865-06

**Authors:** William Procter


### SELECTED.

### REMARKS ON DIALYSIS.

By WILLIAM PROCTER, Jr.

At the meeting of 1862, at Philadelphia, the question, "Is the process of Dialysis applicable in Pharmacy, and if so in what instances may it be employed?" was submitted to me, and not being reported on in 1863 at Baltimore, was continued to the present occasion. As the term "Dialysis" may not be familiar to all present, it has been deemed best to say a few words in regard to it: In the year 1849, Prof. T. Graham, of London, published his views of "liquid diffusion," or the tendency which substances in solution, when brought in contact, have to diffuse themselves into the common menstruum, or into each other. For instance, if a solution of sulphate of zinc, and another of sulphate of magnesia, of the same specific gravity, be carefully placed in contact, one above the other, and left at rest, the law of liquid diffusion will cause the salts to distribute themselves each over the whole menstruum. If only the lower stratum is a saline solution, and the supernatant liquid is water, then, contrary to specific gravity, the salt will diffuse itself into the water above, just as dense gases diffuse themselves into the atmosphere, but in a much slower ratio.

In pursuing this subject afterwards, Prof. Graham made the important discovery that when a hydrated septum, or diaphragm of a gelatinous character, was interposed between the liquid to be diffused and the water into which the diffusion was to take place, many substances could not pass the septum, although in perfect solution, whilst others would pass with greater or less facility. On closely examining into the nature of these two classes of bodies, he found that those which passed the septum were generally crystalline in their nature, or formed crystalline compounds; whilst those which did not pass were amorphous in their structure, and, like gelatin, albumen, starch, etc., did not crystallize. The former he called *crystalloids*, and the latter *colloids.*

It then occurred to Prof. Graham that this law might be rendered subservient to analysis in the separation of one class of bodies from the other; and after many trials, which satisfied him of its value, he called the analysing power of the septum *Dialysis.*

It has long been known that certain animal tissues and porous bodies will allow the passage of one fluid and almost entirely deny the passage of another, when the two fluids are presented at the same time. This quality has been indicated by the terms *exosmose* and *endosmose*, according as the current passed into or out of the vessel of which the tissue formed a part. This quality, which enters largely into the physiological action going on in living vegetables and animals, was supposed to depend more on the nature of the tissues than as influenced by the substances themselves, and it is only since the subject of liquid diffusion has been developed by Graham that more correct views have been entertained.

In the endeavor to render dialysis a practical process, Prof. Graham employed many kinds of tissues, animal membrane of different kinds, thin sheets of gelatin, starch jelly, but none offered so great advantages as the parchment paper of Gaines, which, you are aware, is unsized paper that has been submitted to the action of bihydrated sulphuric acid, and subsequently washed to free it from acid. Parchment paper bears the same relation to crude vegetable fibre (as cotton or flax) that starch granules bear to starch jelly, formed by the rupture and intimate union of those granules. The rationale, given for the action of the hydrated septum, is this: Suppose syrup of gum Arabic mixed with water be placed within the septum—gum being colloidal in its nature has not the power to abstract water from the colloidal septum, whilst the sugar possesses this power, enters the pores of the septum by means of its water, and passes out into the water beyond. The action of parchment paper, and other tissues that act by hydration, can be used only with aqueous solutions, it being yet a desideratum to find a material which shall act equally well with alcoholic and other menstrua.

Mr. Graham's method of dialysing liquids was to use a cylindrical hoop of sheet gutta percha, about two or three inches deep and six inches in diameter, over the lower end of which a sheet of parchment paper is secured by bringing the edges up around the outside of it in a plaited form, and securing them with cord. A larger vessel, capable of holding six or eight times as much liquid, is then filled with water so that the septum may float or be suspended at the surface of the water after it is filled with the liquid to be dialysed, so that the liquids outside of and in the cylinder shall be on a level when the action proceeds from the upper into the lower vessel. This manner of arranging them gives specific gravity in favor of the process, but the action of the septum would also go on if the liquid was at the bottom of the water. The solution of the diffused body is called the *diffusate*, and is always a very dilute solution.

What was then known of this subject was fully developed in an elaborate paper by Prof. T. Redwood, of London, in a lecture before the Pharmaceutical Society. (See *Pharm. Journal*, 515, 1862, and *Amer. Journ. Pharm.*, July, 1862.) The most important points in question are the applicability of the process to the purposes of pharmacy, and pharmaceutical chemistry and toxicology. Heretofore it has been employed in toxicological analysis with the promise of great success, as in dialysing the crude contents of the stomach of a poisoned animal, containing, for instance, arsenious acid, corrosive sublimate, strychnia or morphia. Mr. Redwood has pointed out its probable use in isolating proximate principles in sufficient quantity to be employed by the manufacturer, but heretofore no septum has been found sufficiently active to abridge the time and increase the strength of solutions, to bring the process within the limits of a just economy.

One of the best practical applications of this process is that suggested by Mr. Whitelaw, (*Pharm. Journal*, April, 1864,) of dialysing meat brine to remove its salt, and isolate its extractive material in a fresh state, so as to get extract of meat, which can be used in soup with great nutritive power; and when added to the salt meat restoring it to the value of fresh meat in composition.

The same writer suggests (*Chemical News*, May 28, 1864,) that salt meat may, in the same way, be rendered fresh by being enclosed with its brine in a sack of skin and soaked in water.

